# Berberine derivatives reduce atherosclerotic plaque size and vulnerability in apoE^−/−^ mice

**DOI:** 10.1186/s12967-014-0326-7

**Published:** 2014-11-26

**Authors:** Junwen Chen, Jiatian Cao, Lu Fang, Bo Liu, Qing Zhou, Yinggang Sun, Yue Wang, Yigang Li, Shu Meng

**Affiliations:** Department of Cardiology, Xinhua Hospital Affiliated to Shanghai Jiaotong University, School of Medicine, Shanghai, China; Department of Cardiology, Shanghai Ninth People’s Hospital Affiliated Shanghai Jiaotong University, School of Medicine, Shanghai, China; Vascular Pharmacology Laboratory, Baker IDI Heart and Diabetes Institute, Melbourne, Australia

**Keywords:** Berberine derivatives, EMMPRIN, Inflammation, Atherosclerosis, Plaque stability

## Abstract

**Background and aims:**

Our previous *in vitro* and clinical work has demonstrated anti-inflammatory effects of berberine (BBR), but the clinical application of BBR is limited by its poor bioavailability. Derivatives of BBR have been suggested to have enhanced bioavailability compared to BBR. In this study, we tested whether BBR derivatives, compared with BBR, had superior beneficial effects on atherosclerotic plaques in apoE^−/−^ mice, and defined possible molecular mechanisms underlying such effects.

**Methods:**

Macrophages were pretreated with BBR and its derivatives, dihydroberberine (dhBBR) and 8,8-dimethyldihydroberberine (Di-MeBBR), before incubation with oxLDL. Cell surface EMMPRIN expression was measured by flow cytometry and Western blotting, and phospho-(p)-p38, p-JNK, nuclear NFκB p65, and phospho-p65 were measured by Western blotting. ApoE−/− mice fed with the Western diet for 16 weeks were treated with BBR, dhBBR and Di-MeBBR 16 weeks. Aortic atherosclerotic lesion size, plaque matrix proteins, and EMMPRIN and other inflammatory factors were measured using Oil Red O Staining, Masson’s trichromestaining and immunohistochemical staining and real-time PCR.

**Results:**

Compared with BBR, dhBBR and Di-MeBBR significantly reduced EMMPRIN expression, which was associated with a greater inhibition of p-p38, p-JNK, nuclear NFκB p65 and phospho-p65 induced by oxLDL in macrophages. dhBBR and Di-MeBBR, but not BBR, reduced atherosclerotic plaque size and improved plaque stability indicated by increased α-smooth muscle actin and collagen content, and thicker fibrous caps. dhBBR and Di-MeBBR reduced expression of EMMPRIN, CD68, and NFκB p65, and Di-MeBBR also reduced expression of matrix metalloproteinase-9, intercellular adhesion molecule-1, and vascular cell adhesion molecule-1 in aortic plaques.

**Conclusions:**

These results have demonstrated that BBR derivatives, dhBBR and Di-MeBBR, are superior to BBR in inhibiting inflammation and reducing plaque size and vulnerability.

## Background

Atherosclerosis is a chronic inflammatory disease affecting large and medium-sized arteries throughout the body [1,2]. In the early course of the disease, monocytes adhere to dysfunctional vascular endothelium and migrate into the subendothelial layer of the intima where they differentiate into macrophages. These macrophages transform into foam cells when the subendothelial space is enriched with atherogenic lipoproteins [3]. Foam cells aggregate to form the atheromatous core of the atherosclerotic plaques. Macrophages and foam cells secret a variety of inflammatory factors, which attract more monocytes to infiltrate into the subendothelial space, propagate inflammatory response and subsequently advance atherosclerotic plaques [4]. Macrophage and foam cells also express several matrix metalloproteinases (MMPs), such as MMP-9 and extracellular matrix metalloproteinase inducer (EMMPRIN) [5,6], which, in turn, contribute to vulnerability of atherosclerotic plaques [7,8]. A plaque with a large lipid core and covered by a thin fibrous cap is at a higher risk for rupture [9]. The ruptured fibrous cap is known to be rich in macrophages that produce MMPs, thus digesting extracellular matrix and weakening the fibrous cap. The rupture of an atherosclerotic plaque followed by thrombus formation leads to myocardial infarction, stroke, and death [10]. Therefore, reducing atherosclerotic plaque size and preventing the rupture of vulnerable plaques are essential to preventing emergency medical conditions. In view of the important roles of MMP-9 and EMMPRIN in vulnerability of atherosclerotic plaques, MMP-9 and EMMPRIN are potential targets for therapeutic inventions to inhibit plaque rupture and acute medical conditions.

Berberine (BBR) can be isolated from many different medicinal herbs, such as Berberis, Phellodendron amurense (Huang Po), Coptis chinensis (Huang Lian) [11]. Our previous studies demonstrated that BBR markedly inhibited both mRNA and protein levels of EMMPRIN and MMP-9 in phorbol myristate acetate (PMA)- induced macrophages [12,13]. We further demonstrated that BBR treatment for 30 days, as an adjunct therapy, reduced serum levels of MMP-9, intercellular adhesion molecule (ICAM)-1 and vascular cell adhesion molecule (VCAM)-1 in patients with acute coronary syndrome (ACS) following percutaneous coronary intervention when compared with standard therapy alone [14]. However, whether BBR reduces plaque size and improves plaque stability in animal models is yet to be explored. In addition, several studies have shown that BBR derivatives, such as dihydroberberine (dhBBR) and 8, 8-dimethyldihydroberberine (Di-MeBBR), are more biologically available compared with BBR [15,16], suggesting that BBR derivatives may have greater anti-inflammatory and anti-atherosclerotic effects than BBR.

Therefore, the aims of this study are: 1) to evaluate the inhibitory effects of BBR derivatives on EMMPRIN expression in macrophages and foam cells, in comparison with BBR; 2) to explore whether BBR and its derivatives have *in vivo* anti-atherosclerotic efficacy in apolipoprotein E knock-out (apoE−/−) mice; 3) to study signaling pathways contributing to their anti-atherosclerotic effects.

## Methods

### Cell experiments

Cells from a human monocytic cell line (THP-1 cells, obtained from the American Type Culture Collection) were cultured at a density of 10^6^/mL in RPMI-1640 medium (Hycolone, USA) supplemented with 10% FBS, and 100 U/mL penicillin/streptomycin solution. To induce differentiation of monocytes into macrophages, THP-1 cells were cultured in 100 nM PMA (Calbiochem) for 48 h as previously described [17]. PMA-induced macrophages (2 × 10^6^/mL) were pretreated with BBR (25 μM), or Di-MeBBR (25 μM), dhBBR (25 μM) or thBBR (tetrahydroberberine, 25 μM) for 1 h prior to incubation with oxLDL (50 μg/mL) for the indicated times for different assays. Then, flow cytometry analysis and Western blotting were performed.

### Flow cytometr*y*

The expression of EMMPRIN on the cell surface was determined by flow cytometry. Cells were collected, incubated with FcR blocking reagent and stained directly with a fluorescein isothiocyanate (FITC)-conjugated anti-EMMPRIN antibody (BD, USA) according to the manufacturer’s instruction. An isotype control (BD, USA) was included in each experiment to identify background staining. Samples were analyzed on a BD FACSCalibur flow cytometer (Becton Dickinson, USA).

### Protein isolation and Western blotting analysis

Protein isolation and Western blotting analysis of cell lysates were performed as previously described [12,13]. For the analysis of nuclear NFκB p65, NE-PER nuclear and cytoplasmic extraction reagents (Pierce, USA) were used to separate cytoplasmic and nuclear fractions. Membranes were first probed with primary antibodies for phospho-ERK, ERK, phospho-p38, p38, phospho-JNK, JNK, NFκB p65, phospho-p65 (Ser536), EMMPRIN, and GAPDH (Cell Signaling Technology, USA), and lamin B (Sigma-Aldrich, USA), and then incubated with anti-rabbit or anti-mouse secondary antibodies (Cell Signaling Technology, USA), followed by incubation with antibody labeled with far-red fluorescent Alexa Fluor 680 dye. All signals were detected by the Odyssey Infrared Imaging System (LI-COR, USA) and data were normalized by GAPDH or lamin B levels (for NFκB p65).

### Animal experiments

**Figure 1:**
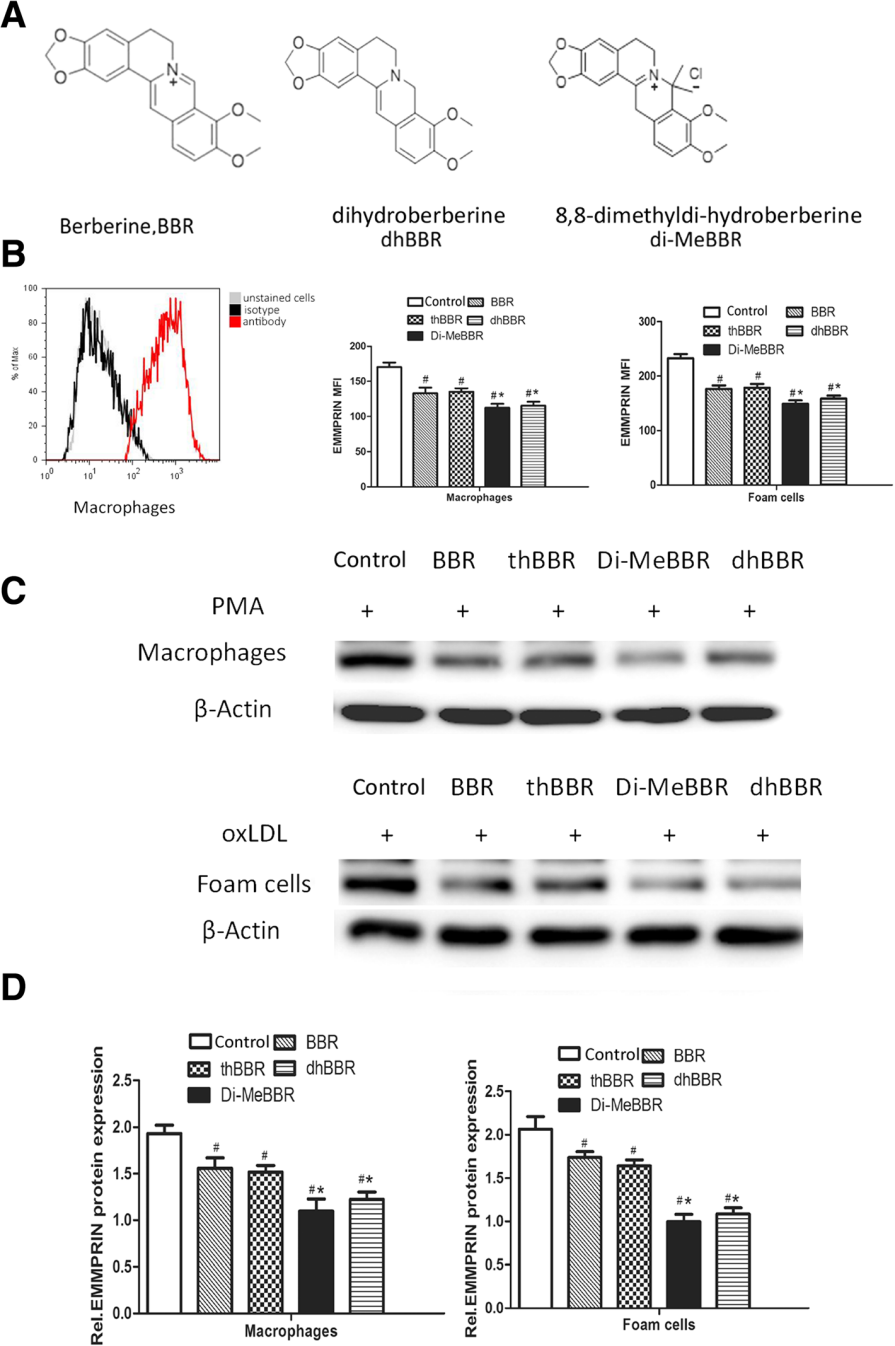
**Molecular structures of berberine and its derivatives and their effects on EMMPRIN expression. (A)** Molecular structures of berberine (BBR) and its derivatives, dihydroberberine (dhBBR), and 8,8-dimethyldi-hydroberberine (di-MeBBR). **(B-D)** The effects of BBR and its derivatives on EMMPRIN expression was monitored by measuring the induction of EMMPRIN via flow cytometry **(B)** and Western blotting **(C, D)**. PMA-induced macrophages were treated with BBR or its derivatives for 1 h (macrophages), or pre-treated with BBR or its derivatives for 1 h prior to incubation with oxLDL (50 μg/mL) for 24 h (foam cells). The expression of EMMPRIN was detected by flow cytometric analysis. *P < 0.05 vs. BBR group. #*P* < 0.05 vs. untreated control group. thBBR: tetrahydroberberine.

All animal experiments were carried out in accordance with the National Institutes of Health Guide for the Care and Use of Laboratory Animals. All methods were approved by the Ethics Committee of Shanghai Jiaotong University School of Medicine. Male apoE−/− mice were obtained from the Animal Research Center of Beijing University. 12-week-old mice (n = 20 per group) were fed with the Western diet (32% fat, 1% cholesterol; Shanghai Slac Laboratory Animal Co. Ltd) for 16 weeks. Mice were treated with BBR (10 mg/kg/d), Di-MeBBR (10 mg/kg/d) or dhBBR (10 mg/kg/d) orally for 16 weeks. BBR was purchased from Sigma-Aldrich, and Di-MeBBR and dhBBR were synthesized by the Shanghai Institute of Materia Medica (Figure 1A) [15]. A control group received phosphate buffered saline (PBS) alone. Following 16 weeks of treatment, mice fasted overnight and were subsequently euthanized for tissue harvesting. Total cholesterol (TC), triglycerides (TG), low-density lipoprotein cholesterol (LDL-C) and high-density lipoprotein cholesterol (HDL-C) were measured from blood obtained by retro-orbital bleeding. Serum lipids were determined using the Roche Automatic Analyzer modular P800 (Roche Diagnostics GmbH, Mannheim, Germany) according to the manufacturer’s instructions.

### Oil red O staining

To examine lipid and plaque accumulation in the aorta, Oil Red O Staining was performed. Mice were perfused through the left ventricle with isotonic saline and then 4% paraformaldehyde in 0.01 M phosphate buffer (pH 7.4). The aorta samples were collected and processed for Oil Red O staining [18]. Briefly, each aorta was rinsed in distilled water using a syringe with needle, quickly rinsed in 70% 2-propanol and stained for 30 min in the Oil Red O working solution (Sigma-Aldrich), and then rinsed again for 10 s in 70% 2-propanol. After that, the aorta was transferred to double-distilled water. This procedure results in red staining of all regions in which atherosclerotic lesions are present. Images were captured by a Canon lSD780 IS digital camera (Tokyo, Japan) and the lesion area was quantitated using Quantity One analysis system (Version 4.6.2). The lesion area of aortic atherosclerosis was expressed as the percentage of Oil Red O-stained area relative to the surface area of the entire aorta (n = 6). All researchers conducting the data capture and analysis were blinded to group.

### Histological analysis

The aorta (from the arch to abdominal aorta) was used. Serial 6 μm sections (40 sections per mouse) of the aorta were collected for immunohistochemical staining and Masson’s trichrome staining. Immunohistochemistry was used to characterize macrophages (CD68, dilution 1:80, Abcam, Cambridge, UK); α-smooth muscle actin (α-SMA, dilution 1:100, Abcam); EMMPRIN (dilution 1:250, Abcam); MMP-9 (dilution 1:300, Abcam); VCAM-1 (dilution 1:200, Abcam); ICAM-1 (dilution 1:150, Abcam); and NFκB p65 (dilution 1:50, Cell Signaling Technology, Inc.) as previously described [13]. Immunohistochemical staining was visualized using an EnVision kit (DAKO) according to the manufacturer’s instructions. Collagen content in the tissue was assessed by Masson’s trichrome staining, similar to the methods previously described [19]. Images were captured with a light microscope camera system (Olympus, Tokyo, Japan) and analyzed using Quantity One analysis system. The results were expressed as the percentage of stained area to total plaque area [20,21]. Fibrous cap thickness of lesion area was evaluated on the most advanced lesion in each mouse by using α-SMA-stained slides.

### Real-time PCR

Total RNA was isolated from the aorta using Trizol (Invitrogen, USA). Real-time PCR was performed to determine gene expression of EMMPRIN, MMP-9, NFκB p65, ICAM-1, VCAM-1, and CD68. The primer sequences are shown in Table 1. mRNA levels of target genes were normalized to the house keeping gene GAPDH.

**Table 1:** **Taqman primer and probe sequences for real-time PCR**

**Gene**	**Forward primer**	**Reverse primer**	**Probe**
CD68	CCGAATCCTATACCCAATTC	CTTGGTTTTGTTGGGATTCA	TGGAAGAAAGGTAAAGCT
α-SMA	CCTCCAGAACGCAAGTACTC	CTTCGTCGTATTCCTGTTTG	CTGGATCGGTGGCTC
EMMPRIN	CAGAAAACCCACCTGGAAGA	ATAAACCCCTAAGGAATGGA	CAGCCCCTGTTTCT
MMP9	TCAAGGATTGCTCAGAGATT	GCAACCACAGTGAGTGAGTT	CCCAGACTCCTCTCT
NFkB p65	GGCAGTATTCCTGGCGAGAGA	CCAGGGAGATTCGAACTGTT	ATGGCTACACAGGACC
ICAM-1	CCTTAGCAGCTGAACAATCG	TAAGGAGTTGGAACATTTCC	AGGCGGCTCCACC
VCAM-1	GACAAGTCCCCATCGTTGAA	ACCTCGCGACGGCATAATTT	CTGGAATGGTCCCATGAT

α-SMA, α-smooth muscle actin; EMMPRIN, extracellular matrix metalloproteinases inducer; ICAM-1, intercellular adhesion molecule-1; MMP-9, metalloproteinase-9; NFκB, nuclear factor-κB; VCAM-1, vascular cell adhesion molecule-1.

### Statistical analysis

SPSS v17.0 statistical software package was used for data analysis. Results were expressed as mean ± standard deviation. All variables were tested for homogeneity of variance by Levènes test. If data were distributed normally with equal variance, multiple-comparison tests between the groups were performed with one-way ANOVA followed by Student-Newman-Keuls post hoc analysis. Otherwise, multiple-comparison were analysed using nonparametric Kruskal-Wallis one-way ANOVA followed by Bonferroni correction. *P* <0.05 was considered statistically significant (2-sided).

## Results

### *In vitro* studies

#### Effects of BBR and its derivatives on EMMPRIN expression

Since EMMPRIN functions as an upstream regulator of MMPs such as MMP-9 [22,23], we compared the effects of BBR and its derivatives on EMMPRIN expression by flow cytometric analysis and Western blotting. PMA-induced macrophages were treated with BBR or its derivatives for 1 h (macrophages), or pre-treated with BBR or its derivatives for 1 h prior to incubation with oxLDL (50 μg/mL) for 24 h (foam cells). Compared to BBR group, Di-MeBBR and dhBBR groups showed lower EMMPRIN expression in both macrophages and foam cells (Figure 1b-d, *P* < 0.05), but thBBR group did not differ from BBR group. So, we only tested dhBBR and Di-MeBBR for the following investigations in this study.

#### Effects of BBR and its derivative on activation of MAPK pathways

**Figure 2:**
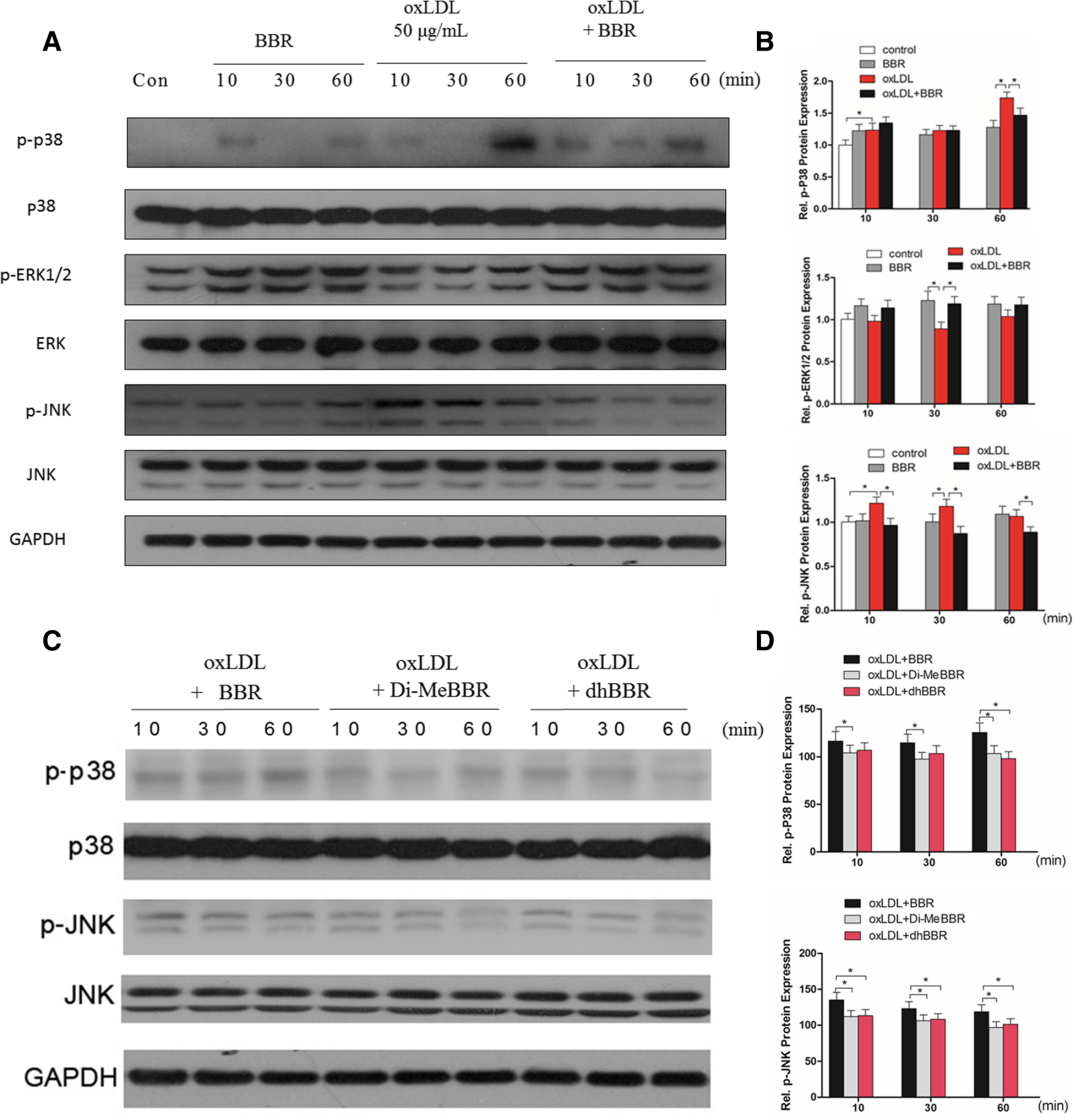
**Effects of berberine and its derivatives on activation of MAPK signaling pathways induced by oxLDL. (A-B)** Cells were pretreated with berberine for 1 h, and then incubated with oxLDL for 10, 30 and 60 min. The cell lysates were collected for Western blotting to measure protein levels of phospho (p)-p38, p-ERK1/2, and p-JNK. **(A)** Representative bands of Western blotting. **(B)** Quantification of p-p38, p-ERK1/2 and p-JNK bands. (C-D)Cells were pretreated with berberine or dhBBR, or Di-MeBBR for 1 h, followed by incubation with oxLDL for 10, 30 and 60 min. The cell lysates were collected for Western blotting to measure phospho (p)-p38 and p-JNK. **(C)** Representative bands of Western blotting. **(D)** Quantification of p-p38, p-ERK1/2 and p-JNK bands.GAPDH expression was used for protein level normalization. *P < 0.05 vs. BBR group.

Our previous studies and other studies suggested that mitogen-activated protein kinase (MAPK) pathways were involved in expression of EMMPRIN, MMP-9 and CD68 and formation of foam cells [12,13,24], so we studied the effects of BBR and its derivatives on phosphorylation of MAPK induced by oxLDL in macrophages. Macrophages were pretreated with BBR and its derivatives for 1 h, and then stimulated with oxLDL for 10, 30 and 60 min. We found that oxLDL increased phosphorylation of p38 and JNK at 10, 30 and 60 min, but it had no effect on ERK1/2 phosphorylation (Figure 2A,B). BBR pretreatment decreased phosphorylation of p38 and JNK induced by oxLDL (Figure 2A, B). Compared to BBR, Di-MeBBR and dhBBR had a greater inhibition of phosphorylation of p38 and JNK (Figure 2C, D).

#### Effects of BBR and its derivative on translocation of NFκB p65 to the nucleus

**Figure 3:**
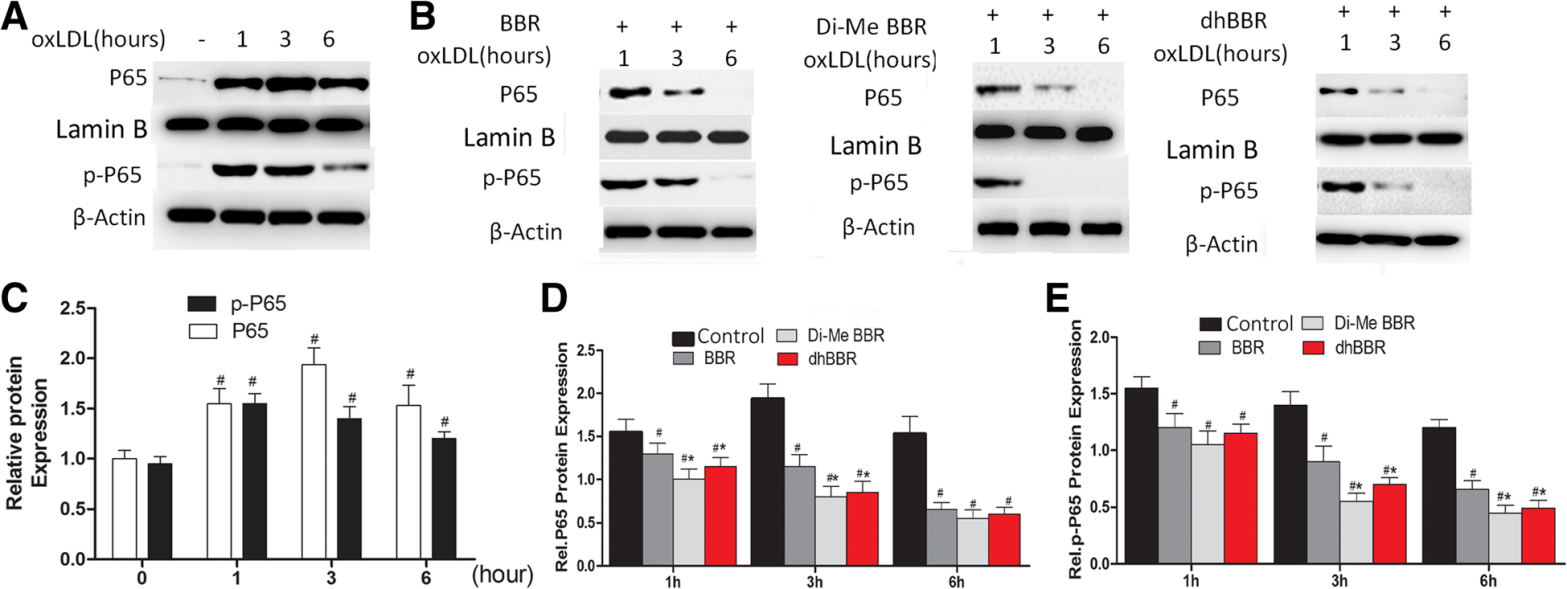
**Comparisons of berberine and its derivatives on activation of MAPK signaling pathways.** Cells were pretreated with berberine or dhBBR, or Di-MeBBR for 1 h, followed by incubation with oxLDL for 1, 3, and 6 h. Nuclear fractions were isolated, nuclear NFκB p65 and phospho-p65 were measured by Western blotting. **(A, C)** Effects of oxLDL on nuclear translocation of NFκB p65 and phospho-p65 expression. **(B, D, D)** Berberine and its derivatives (dhBBR, di-MeBBR) on nuclear translocation of NFκB p65 and phospho-p65 expression. Lamin B and β-Actin was used for protein level normalization. *P < 0.05 vs. BBR group. ^#^ *P* < 0.05 vs. 0 h **(C)** or untreated control group **(D, E)**.

Our previous study showed that BBR also suppressed MMP-9 and EMMPRIN expression by inhibiting NFκB p65 activation in oxLDL-stimulated macrophages [12]. In this study, we examined whether BBR derivatives had greater impacts on the activation of NFκB p65. Macrophage were pretreated with BBR, Di-MeBBR and dhBBR, and then stimulated with oxLDL for 1, 3 and 6 h. NFκB p65 and phosho-p65 in nuclear fractions of macrophages were significantly increased by stimulation with oxLDL for 1, 3, and 6 h (Figure 3A, C). p65 peaked at 3 h and phosphor-p65 peaked at 1 and 3 h. Compared to BBR, Di-MeBBR and dhBBR blocked the nuclear translocation of NFκB p65 and phosho-p65 more significantly (Figure 3B, D, E).

### *In vivo* studies

#### Body weight and serum lipids

At the time of sacrifice, there was no difference in body weight between the experimental and control mice (*P* >0.05). Compared with the control group, TC levels were significantly lower in Di-MeBBR and BBR group. LDL-C levels were significantly lower in BBR, dhBBR and Di-MeBBR group (Table 2). TG and HDL-C levels did not differ among groups.

**Table 2:** **The effects of BBR and its derivatives on weight and lipid profiles**

	**Weight (g)**	**TG (mmol/L)**	**TC (mmol/L)**	**HDL-C (mmol/L)**	**LDL-C (mmol/L)**
Control	34.2 ± 0.5	5 ± 1.1	22.9 ± 1.5	1.6 ± 0.25	5.9 ± 1.12
BBR	33.4 ± 0.7	3.9 ± 0.8	17.3 ± 2.1*	1.7 ± 0.31	4.8 ± 1.2*
dhBBR	32.1 ± 1.2	4.2 ± 0.7	21.6 ± 1.8	1.6 ± 0.29	5.1 ± 1.3*
Di-MeBBR	33.6 ± 1.7	4.4 ± 0.9	18.9 ± 1.3*	1.8 ± 0.32	4.5 ± 0.9*

Data are shown as means ± SD. *P <0.05 vs. control. TG, total triglycerides; TC, total cholesterol; HDL-C, high-density lipoprotein cholesterol; LDL-C, low-density lipoprotein cholesterol; BBR, berberine; dhBBR, dihdroberberine; Di-MeBBR, 8,8-dimethyldihydroberberine.

#### Aortic atherosclerotic lesion area

**Figure 4:**
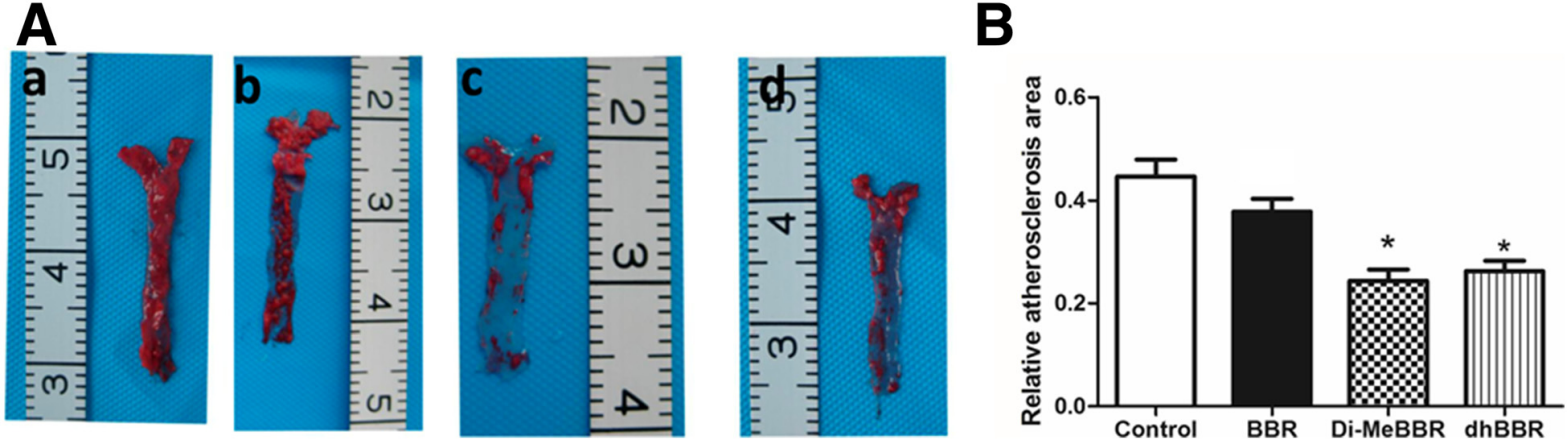
**Effects of berberine and its derivatives on atherosclerotic plaque area.** ApoE−/− mice were fed with the Western diet for 16 weeks and then treated with BBR (b) or its derivatives, Di-MeBBR (c) and dhBBR (d) for 16 weeks. Mice receiving PBS served as the control group (a). **(A)** The atherosclerotic lesions were stained by Oil RedO. **(B)** Plaque area was quantified as the percentage of Oil Red O-stained area to total aortic surface area.*P < 0.05 vs. the control group.

The lesion area was significantly smaller in mice treated with dhBBR and Di-MeBBR compared with control mice (*P* < 0.05). There was no difference in lesion area between BBR group and the control group (Figure 4A, B).

#### Expression of CD68, EMMPRIN, MMP-9, NFκB, ICAM-1 and VCAM-1

**Figure 5:**
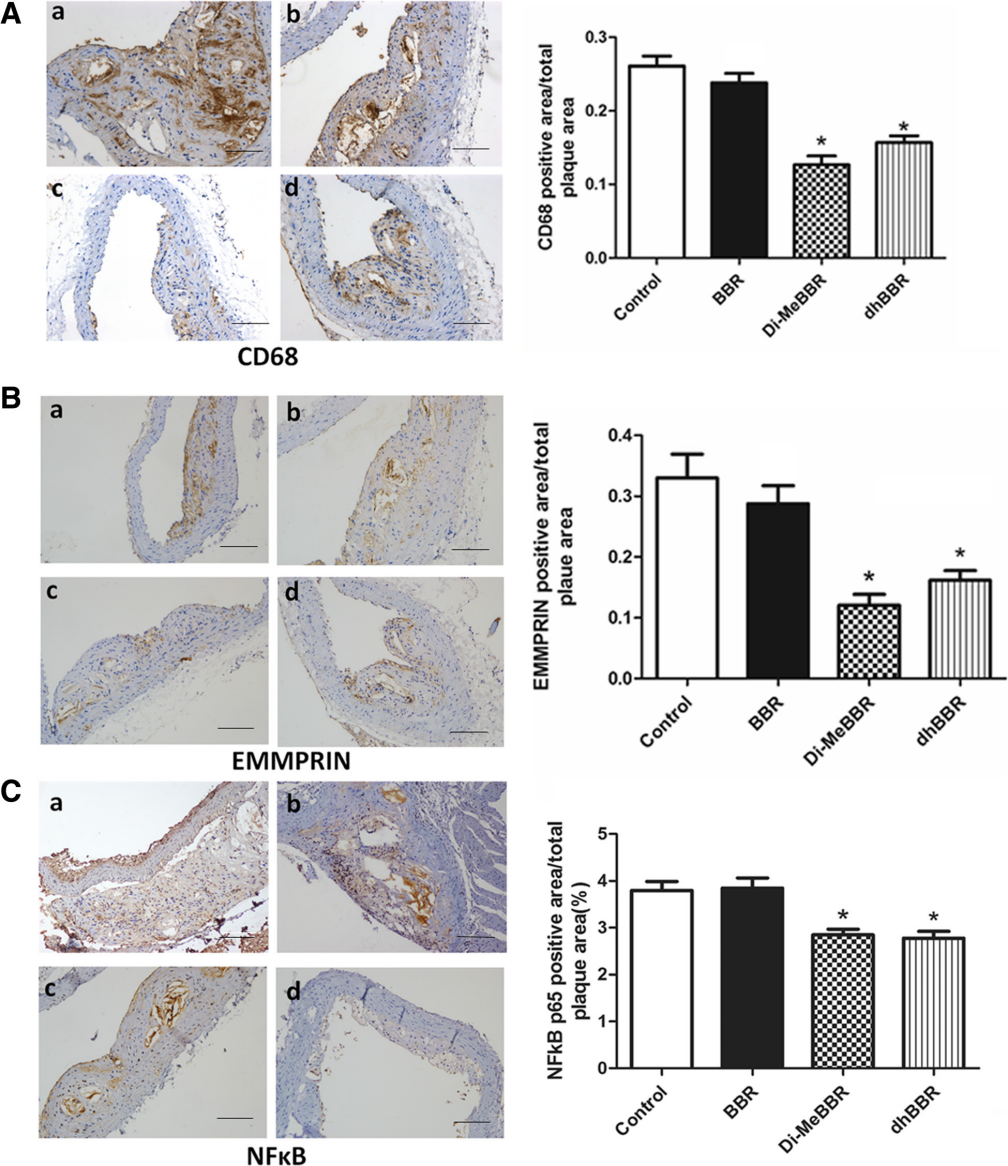
**Effects of berberine and its derivatives on CD68, EMMPRIN, and NFκB p65 protein expression in atherosclerotic plaques.** ApoE−/− mice were fed with the Western diet for 16 weeks and then treated with BBR (b) or its derivatives, Di-MeBBR (c) and dhBBR (d) for 16 weeks. Mice receiving PBS served as the control group (a). Mouse aorta sections were collected. Representative immunostaining images and quantification of CD68 **(A)**, EMMPRIN **(B)** and NFκB p65 **(C)** in atherosclerotic plaques. Scale bar represents 100 μm. *P < 0.05 vs. the control group.

**Figure 6:**
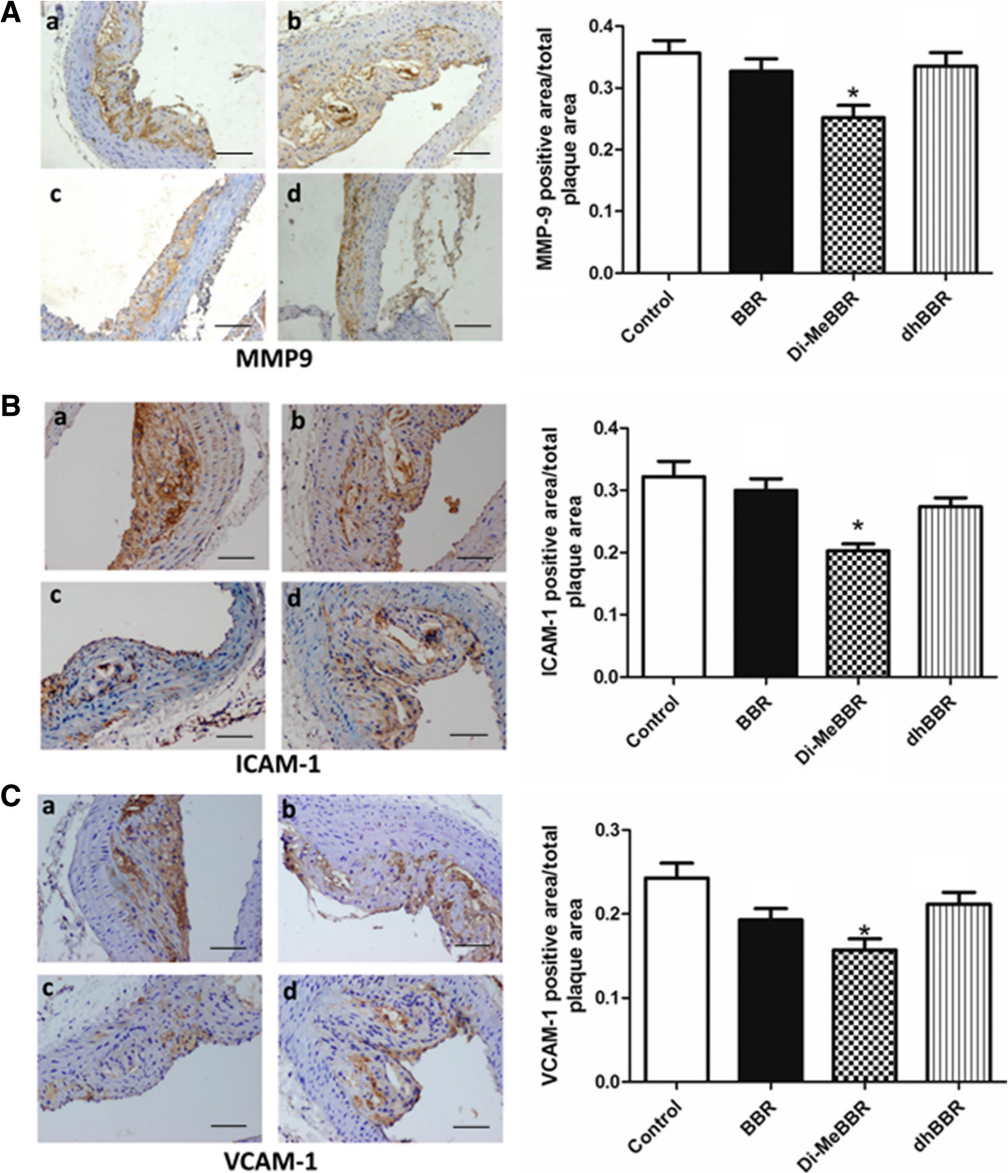
**Effects of berberine and its derivatives on protein expression of inflammatory mediators in atherosclerotic plaques.** ApoE−/− mice were fed with the Western diet for 16 weeks and then treated with BBR (b) or its derivatives, Di-MeBBR (c) and dhBBR (d) for 16 weeks. Mice receiving PBS served as the control group (a). Mouse aorta sections were collected. Representative immunostaining images and quantification of MMP-9 **(A)**, ICAM-1 **(B)** and VCAM-1 **(C)** in atherosclerotic plaques. Scale bar represents 100 μm. *P < 0.05 vs. the control group.

**Figure 7:**
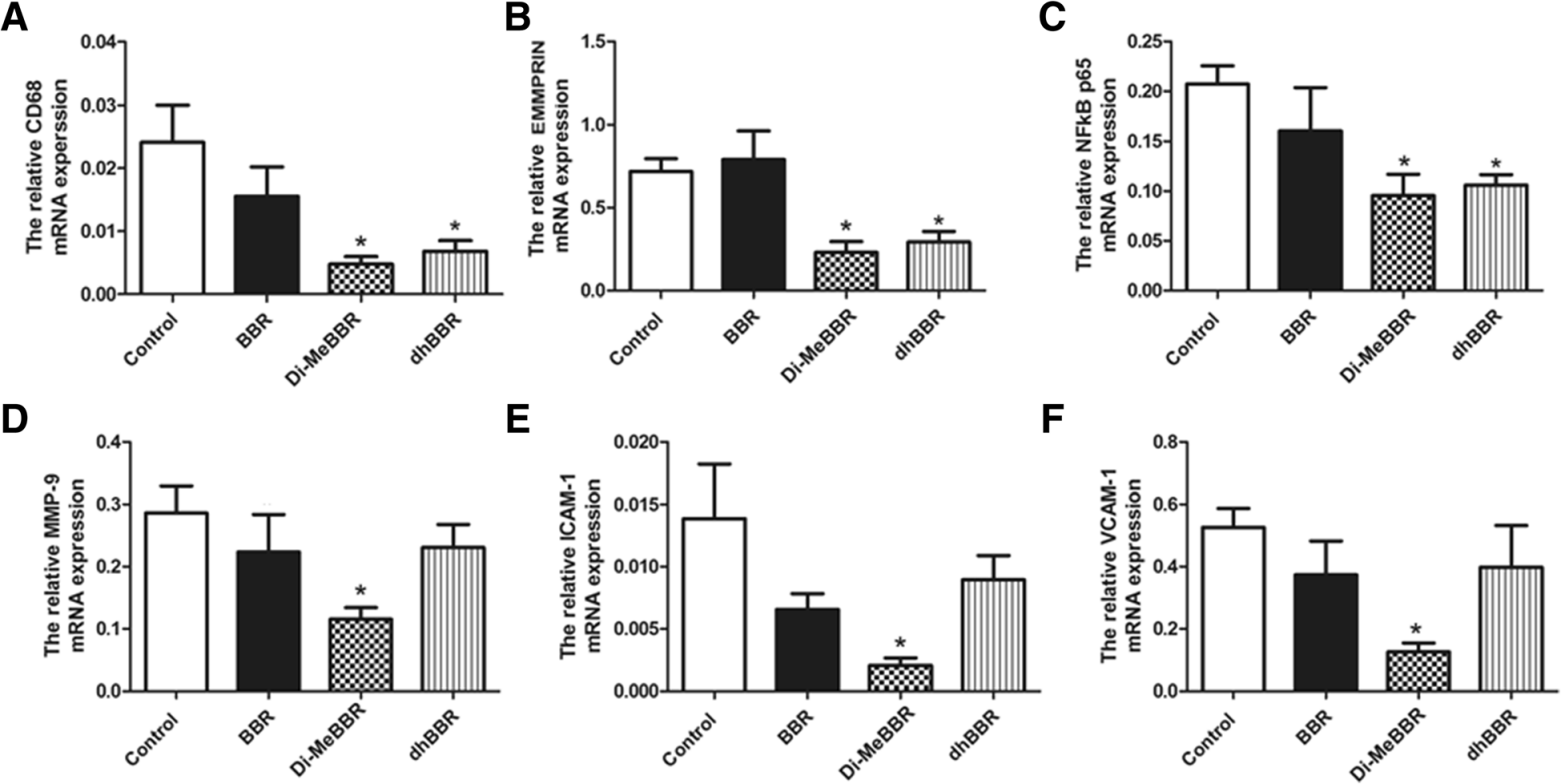
**Effects of berberine and its derivatives on gene expression of inflammatory mediators in atherosclerotic plaques.** ApoE−/− mice were fed with the Western diet for 16 weeks and then treated with BBR or its derivatives (dhBBR and Di-MeBBR) for 16 weeks. Real-time PCR was performed to determined mRNA expression of CD68 **(A),** EMMPRIN **(B)**, NFkB p65 **(C)**, MMP-9 **(D)**, ICAM-1 **(E)**, VCAM-1 **(F)** in the aorta. *P < 0.05 vs. the control group.

As macrophage accumulation in the arterial wall contributes to atherosclerotic lesion development, we investigated expression of the macrophage marker, CD68, by immunohistochemistry in atherosclerotic plaques (Figures 5 and 6) and real-time PCR in the aorta (Figure 7). We also measured EMMPRIN, MMP-9, NFκB p65, and two key cell adhesion molecules (ICAM-1, VCAM-1) by immunohistochemistry (Figures 5 and 6) and real-time PCR (Figure 7). Compared to vehicle control, Di-MeBBR significantly reduced expression of CD68, EMMPRIN, NFκB p65, MMP-9, ICAM-1 and VCAM-1, whereas dhBBR significantly reduced expression of CD68, EMMPRIN, and NFκB p65, without significant effect on expression of MMP-9, ICAM-1 and VCAM-1. BBR did not have effect on the expression of any of these molecules (Figures 5, 6 and 7).

### α-SMA and collagen in atherosclerotic plaques

**Figure 8:**
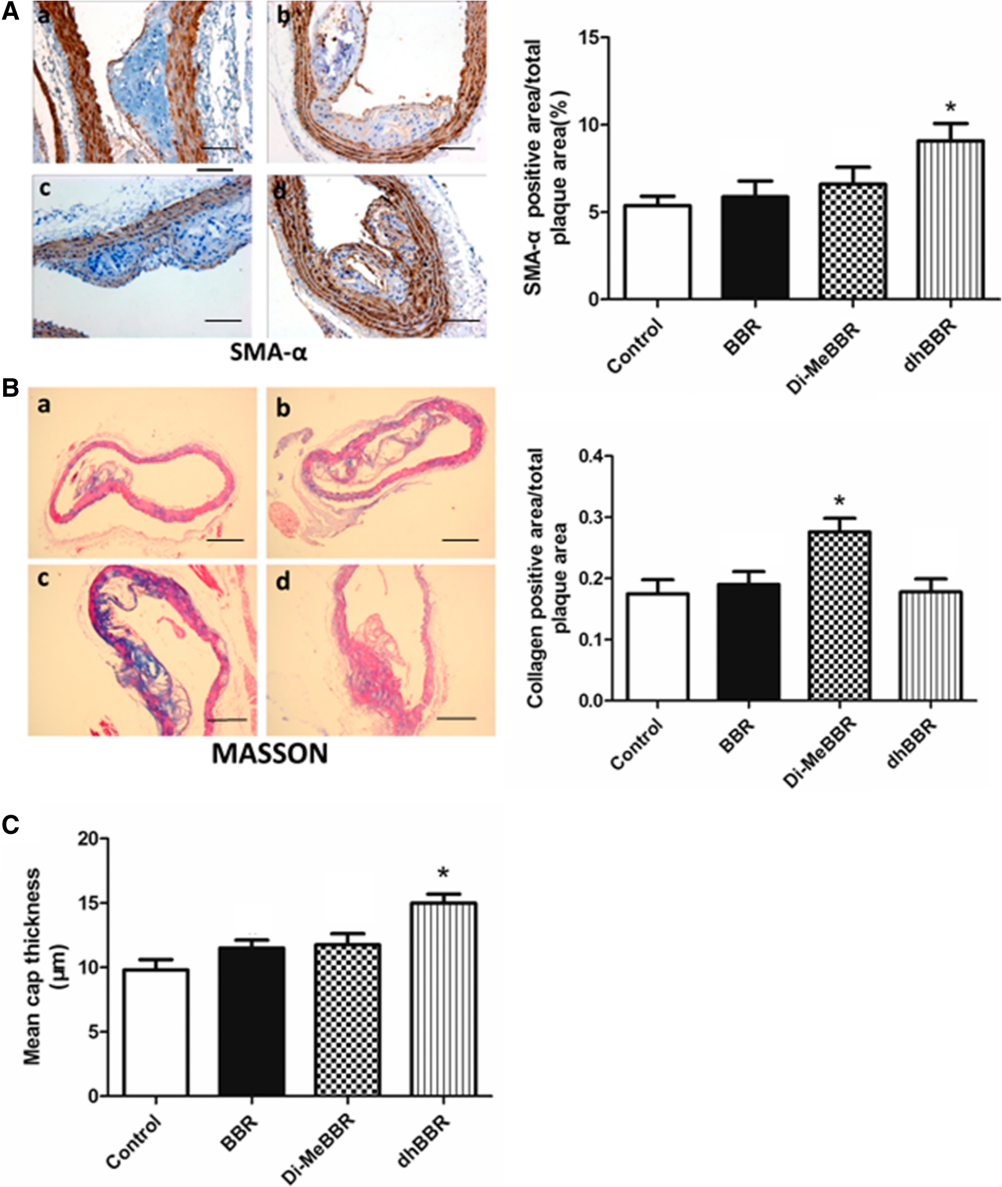
**Effects of berberine and its derivatives on α-SMA and collagen content and fibrous cap thickness in atherosclerotic plaques.** ApoE−/− mice were fed with the Western diet for 16 weeks and then treated with BBR **(B)** or its derivatives, Di-MeBBR **(C)** and dhBBR **(D)** for 16 weeks. Mice receiving PBS served as the control group **(A)**. Mouse aorta sections were collected. Immunohistochemistry and Masson’s trichrome staining were performed to determined the positive area of α-SMA,scale bar represents 100 μm **(A)** and collagen,scale bar represents 500 μm **(B)** content, respectively. The mean fibrous cap thickness was measured **(C)**. *P < 0.05 vs. the control group.

We further determined α-SMA and collagen levels in plaques using immunohistochemistry and Masson’s trichrome staining, respectively. Compared to vehicle control group, Di-MeBBR group had more collagen deposition (Figure 8B), while dhBBR group showed increases in α-SMA content (Figure 8A) and fibrous cap thickness (Figure 8C). However, BBR had no effect on collagen, α-SMA and fibrous cap thickness.

## Discussion

Our previous studies have found that BBR inhibits MMP-9 and EMMPRIN expression in macrophages [12] and also decreases plasma MMP-9, ICAM-1 and VCAM-1 in patients with ACS [14]. However, the clinical use of BBR is limited by its poor bioavailability. In the present study, we evaluate anti-atherosclerotic effects of BBR derivatives (known to have enhanced bioavailability) *in vitro* and *in vivo*, in comparison with BBR. Our *in vitro* study showed that BBR and its derivatives, dhBBR and Di-MeBBR, decreased EMMPRIN expression and inhibited phosphorylation of p38 and JNK and nuclear translocation of NFκB p65 induced by oxLDL in macrophages, with dhBBR and Di-MeBBR showing greater effects in comparison with BBR. Our *in vivo* study demonstrated that dhBBR and Di-MeBBR reduced aortic atherosclerotic lesion size, improved plaque stability (increased α-SMA and collagen content, and increased fibrous cap thickness), and decreased EMMPRIN, MMP-9, CD68, ICAM-1, VCAM-1 and NFκB p65 expression when compared with vehicle control in apoE−/− mice fed with the Western diet. However, BBR at the same dosage did not show beneficial effect on atherosclerosis in apoE−/− mice. Take together, our results have shown that dhBBR and Di-MeBBR have better anti-inflammatory effects than BBR, and dhBBR and Di-MeBBR, but not BBR, reduce atherosclerotic plaque size and improve plaque stability in apoE−/− mice. Therefore, dhBBR and Di-MeBBR have therapeutic advantages over BBR as an attractive adjunct therapy in patients with atherosclerosis.

Although our previous *in vitro* and clinical studies have shown that BBR has anti-inflammatory effects, we have not explored whether BBR reduces atherosclerosis in animal models. In the present study, we have demonstrated that BBR derivatives at 10 mg/kg/d not only reduce plaque size, but also improve plaque stability in apoE−/− mice, however BBR at the same dosage had no significant anti-atherosclerotic effect. One of the earliest events in atherogenesis is the adhesion of monocytes to the vascular endothelium, which is mainly mediated by ICAM-1 and VCAM-1 [25]. Following initial adhesion, monocytes subsequently infiltrate into the subendothelial space and differentiate into macrophages. Macrophages and foam cells contribute to the initiation and development of atherosclerosis by forming fatty streak and secreting a variety of inflammatory mediators [4]. In this study, we found that both dhBBR and Di-MeBBR reduced CD68 expression in atherosclerotic plaques in apoE−/− mice, indicating reduced infiltration of macrophages. Di-MeBBR also decreased ICAM-1 and VCAM-1 expression in atherosclerotic plaques, thus inhibiting the initial step of monocyte recruitment. Although our previous clinical research showed BBR reduced plasma ICAM-1 and VCAM-1 in patients with ACS [14], BBR had no effect on CD68, ICAM-1 and VCAM-1 in apoE−/− mice. Macrophages also contribute to plaque rupture by secreting MMPs that degrade extracellular matrix proteins and weaken the fibrous cap [26,27]. Among various MMP species, MMP-9, mainly deriving from macrophages and foam cells, plays an important role in plaque rupture and ACS [7,27,28]. EMMPRIN is an upstream inducer of MMPs [6,22,23], and it induces MMP-9 synthesis in a paracrine or autocrine manner [29]. We previously found that BBR inhibited MMP-9 and EMMRIN expression in macrophages. In this study, we further showed that dhBBR and Di-MeBBR, compared with BBR, had greater inhibitory effects on EMMPRIN expression in both macrophages stimulated with oxLDL and atherosclerotic plaques in apoE−/− mice. Di-MeBBR also inhibited MMP-9 expression in atherosclerotic plaques. Take together, BBR derivatives reduce plaque size and improve plaque stability through suppressing both the accumulation of macrophages and the production of EMMPRIN and MMP-9 by macrophages.

The protective roles of BBR derivatives against atherosclerosis are likely mediated by NFκB and MAPK signaling pathways. NFκB pathway is a major transcription factor that regulates gene expression of a wide variety of inflammatory mediators including cell adhesion molecules, chemokines, cytokines, and MMPs [30], which are key players in the pathogenesis and development of atherosclerosis and its complications. There are a variety of stimuli which can activate NFκB including vascular injury, oxLDL, and cytokines etc. [30]. One of the key steps in activating NFκB pathway is the stimulation of the IκB kinases [31]. In unstimulated cells, NFκB canonical signaling pathway p65 is localized predominantly in the cytoplasm and remains inactivated through binding with its inhibitory protein, IkB-α. Upon oxLDL stimulation, IκB-α is phosphorylated and subsequently degraded, which allows p65 to be liberated from IkB-α. NFκB is then translocated into the nucleus where it binds to a specific sequence in the promoter of target genes, resulting in increased expression of target genes [30]. NFκB may also be activated by MAPK signaling pathways [32]. MAPK signaling pathways are known to play an important role in the pathogenesis of cardiac and vascular disease including atherosclerosis [33]. Studies have shown that activation of JNK and p38, but not ERK1/2, is required for foam cell formation [24,34,35]. Activation of JNK and p38 also induces gene expression of inflammatory mediators such as adhesion molecules and MMPs [36-38]. Our previous study found that BBR inhibited activation of NFκB and p38 induced by oxLDL in macrophages [12,13]. The current study further demonstrated that dhBBR and Di-MeBBR exhibited greater inhibition on activation of NFκB and MAPK (p38 and JNK) in oxLDL-stimulated macrophages and on expression of NFκB p65 in atherosclerotic plaques in apoE−/− mice. BBR derivatives thus exert anti-inflammatory and anti-atherosclerotic effects by targeting NFκB and MAPK (p38 and JNK) signaling pathways.

Superiority of BBR derivatives over BBR is likely due to their enhanced bioavailability. The structure of BBR is extremely flat, which limits its absorption across the intestinal epithelia. In BBR derivatives, the D ring side chain is filled with various substituents, alternating the double bond of ring C and attaching different substituents at the 13-position and other positions [16]. Changing to dhBBR opens up the structure, making it more amenable to uptake, which is substantiated by pharmokinetic data [15]. Enhanced bioavailability of dhBBR leads to improved *in vivo* efficacy on metabolism [15]. Once absorbed, dhBBR is rapidly converted to BBR, so dhBBR is likely the active moiety. However, dhBBR can also be converted to BBR in the stomach, which would hinder *in vivo* absorption and reduce its bioavailability [15]. Blocking aromatization of dhBBR 8,8-disubstitution with an alkyl group optimizes dhBBR into more aqueous soluble and acid stable 8,8-dialkyldihydroberberine hydrochlorides. 8,8-Dimethyl-13,13a- dihydroberberine (Di-MeBBR) has been identified as a promising derivative. With improved aqueous solubility and acid stability, Di-MeBBR has significantly higher bioavailability without conversion back to BBR *in vivo*. Di-MeBBR improves glucose and lipid metabolism in diet-induced obese mice at a relatively low dosage and has been shown to be more effective than dhBBR in db/db mice [16]. In the present study, overall, BBR derivatives have better anti-inflammatory effects than BBR, and reduce atherosclerotic plaque size and instability in apoE−/− mice. There are some differences between Di-MeBBR and dhBBR in terms of improving plaque stability. Compared to vehicle control group, Di-MeBBR group has higher collagen content, whereas dhBBR group has higher α-SMA content and thicker fibrous cap. We have shown that Di-MeBBR, but not dhBBR or BBR, significantly reduces MMP-9 gene and protein expression in apoE−/− mice, which could lead to reduced collagen degradation and higher collagen content in Di-MeBBR group. α-SMA is primarily produced by smooth muscle cells. Whether dhBBR has the effect on smooth muscle cells needs further investigation. In addition, in previous studies [12,13] and this study, we pretreated cells with BBR and its derivatives before oxLDL stimulation. Our next study will investigate whether treatment of BBR and its derivatives at the same time of or after oxLDL stimulation will exert similar anti-inflammatory effects.

## Conclusions

Our results demonstrate that BBR derivatives (dhBBR and Di-MeBBR) have better inflammatory and anti-atherosclerotic effects compared with BBR. So, BBR derivatives could have therapeutic advantages over BBR as an adjunct therapy in patients with atherosclerosis.

## Abbreviations

ACS: Acute coronary syndrome
α-SMA: α-smooth muscle actin
BBR: Berberine
dhBBR: Dihdroberberine
Di-MeBBR: 8,8-dimethyldihydroberberine
EMMPRIN: Extracellular matrix metalloproteinase inducer
HDL-C: High-density lipoprotein cholesterol
ICAM-1: Intercellular adhesion molecule-1
LDL-C: Low-density lipoprotein cholesterol
MAPK: Mitogen-activated protein kinase
MMPs: Metalloproteinases
NFκB: Nuclear factor-κB
TC: Total cholesterol
TG: Total triglycerides
VCAM-1: Vascular cell adhesion molecule-1
